# Secular trend in interobserver agreement of VIA diagnosis for cervical cancer screening in Nigeria

**DOI:** 10.1371/journal.pone.0208531

**Published:** 2018-12-06

**Authors:** Eileen O. Dareng, Yinka Olaniyan, Michael K. Odutola, Sally N. Adebamowo, Ayotunde Famooto, Richard Offiong, Kayode Obende, Stephen A. Adewole, Peter Achara, Patrick S. Dakum, Clement A. Adebamowo

**Affiliations:** 1 Department of Public Health and Primary Care, University of Cambridge, Cambridge, United Kingdom; 2 Department of Strategic Information and Research, Institute of Human Virology Nigeria, Abuja, Nigeria; 3 Department of Obstetrics and Gynaecology, National Hospital, Abuja, Nigeria; 4 Department of Epidemiology and Public Health, University of Maryland School of Medicine, Baltimore, Maryland, United States of America; 5 Marlene and Stewart Greenebaum Comprehensive Cancer Center, University of Maryland, Baltimore, Maryland, United States of America; 6 Department of Obstetrics and Gynaecology, University of Abuja Teaching Hospital, Gwagwalada, Abuja, Nigeria; 7 Department of Obstetrics and Gynaecology, Garki Hospital Abuja, Abuja, Nigeria; 8 Department of Obstetrics and Gynaecology, Mother and Child Hospital Ondo, Ondo Nigeria; 9 Department of Obstetrics and Gynaecology, Federal Medical Centre, Keffi, Nigeria; 10 Institute of Human Virology, University of Maryland School of Medicine, Baltimore, Maryland, United States of America; Bharathidasan University, INDIA

## Abstract

**Objective:**

In low resource settings, visual inspection with acetic acid (VIA) by allied health workers, has been suggested as an alternative for cervical cancer screening. However, there are concerns about the objectivity and time to diagnostic concordance with specialists. We evaluated the secular trend in interobserver agreement between nurse providers and a gynecologist/colposcopist over a five-year period.

**Methods:**

Nurses provided VIA screening with digital cervivography to 4,961 participants in five screening clinics from October 2010 to May 2014 in Nigeria in this observational study. Cervigraphs were reviewed at meetings where a gynaecologist/colposcopist made an assessment from the cervigraphs. We used weighted kappa statistics to calculate agreement in diagnosis between nurse providers and the gynecologist/colposcopist; linear regression models to examine overall trend and investigate potential clinic characteristics that may influence agreement; and time series models to characterize month to month variations.

**Results:**

Mean age of participants was 37±8 years. Overall agreement was 0.89 at Site D, 0.78 and 0.73 at Sites A and C respectively, 0.50 for Site E and 0.34 for Site C. The number of trainings attended by nurse providers(β = 0.47,95%CI:0.02–0.93, p = 0.04), high level of engagement by site gynecologists(β = 0.11,95%CI:0.01–0.21,p = 0.04) were associated with increased agreement; while increasing distance from the coordinating site(β = -0.47,95%CI:-0.92–0.02,p = 0.04) was associated with decreased agreement. There were no associations between number of years screening clinics were operational(β = 0.01,95%CI: -0.01–0.03,p = 0.29), cumulative experience of nurse providers(β = 0.04,95%CI:-0.03–0.12,p = 0.19) and agreement. There were no significant increases in weighted kappa statistics over time for all sites considered. Monthly variations were significant for only one of two sites considered in time series models (AR1 term = -0.40, 95%CI:-0.71–0.09,p = 0.01).

**Conclusion:**

Our results showed a lack of objectivity, persistent variation and lack of convergence of diagnostic capabilities of nurse led VIA cervical cancer screening with the diagnostic capabilities of a specialist in a cervical cancer screening program in Nigeria.

## Introduction

Cervical cancer is a major cause of morbidity and mortality, especially in low and middle-income countries (LMIC) [1,2]. In 2015, 526,000 incident cases and 239,000 deaths were estimated to have occurred globally [1]. Up to 85% of these incident cases and deaths occurred in LMIC, where the lifetime odds of developing cervical cancer can be as high as 1 in 24 women compared to 1 in 115 in more developed regions [1, 3]. A historic investment in cytology-based, and more recently HPV DNA test-based population level cervical cancer screening as well as access to care is largely responsible for the lower burden of cervical cancer observed in high income countries [4]. In contrast, most LMICs have not been successful in implementing sustainable and effective cervical cancer screening programs.

Barriers to the successful implementation of cytology or HPV DNA test based cervical cancer screening programs in LMIC include the need for well-trained laboratory technicians; adequate clinical and laboratory infrastructure to support sample collection and processing; and requirements for multiple clinic visits for screening and treatment. In order to address these limitations, screen and treat strategies which are based on visual inspection with acetic acid (VIA) followed by same day ablative treatment implemented by nurses and allied health workers, have been promoted as cost-effective cervical cancer screening methods in LMIC [5]. The World Health Organization (WHO) and the American Society of Clinical Oncology (ASCO) recommend the use of VIA programs with significant investment in provider training and incorporation of stringent quality control measures to evaluate provider performance [5, 6].

Results from studies in India, Latin America and Africa suggest that the sensitivity of VIA is comparable to cytology-based screening strategies [7, 8]. However, other researchers have found VIA to be highly subjective with sensitivity ranging from 41% to 79% even among well-trained practitioners [9]. Incorporation of digital cervicography for immediate and subsequent secondary review has been promoted as a way to improve diagnostic accuracy [10, 11]. In addition to quantifying the level of subjectivity, it is also important for program implementation to ascertain whether VIA providers become more proficient over time as expertise of providers increase. This would help in the design and monitoring of cervical cancer screening programs based on VIA. Nevertheless, there has been no studies of the secular trend in diagnostic accuracy comparing nurse or allied health worker providers and specialist physicians in order to ascertain minimum duration of training required before providers become independent and whether the diagnostic capabilities of providers converge with that of highly skilled gynecologists/colposcopists over time.

In this study, we evaluate the secular trend in interobserver agreement between diagnosis made by trained nurse providers and a gynecologist/colposcopist over a 44-month period (October 2010 –May 2014) in a VIA and digital cervicography based cervical cancer screening program in Nigeria.

## Materials and methods

### Study population

Between October 2010 and May 2014, 4,961 women who were 18 years or older, participated in VIA based screen and treat cervical cancer screening program in five health facilities–National Hospital, Abuja (NHA); University of Abuja Teaching Hospital (UATH); Garki Hospital Abuja (GHA); Federal Medical Center, Keffi (FMCK) and Mother and Child Hospital, Ondo (MCHO) in Nigeria. Screening activities at these clinical sites were coordinated from a central office in Abuja, Nigeria. Three of the sites were located close to the coordinating office (NHA, UATH and GHA) while the other were located in the North Central (FMCK) and South Western (MCHO) regions of the country respectively. The distance from these sites to the coordinating office were 70 kilometers (km) for FMCK and 560km for MCHO.

We trained nurses to perform a physical examination including a detailed abdomino-pelvic examination. This was followed by VIA and digital cervicography. Results of VIA examinations were recorded as either positive, negative, uncertain or suspicious of cancer according to the International Agency for Research Against Cancer (IARC) guidelines [12]. Women who were VIA positive were treated with thermocoagulation, if eligible or referred for colposcopy, biopsy and subsequent treatment at the gynecology clinic if considered ineligible for thermocoagulation. Eligibility criteria for treatment were: complete visualization of acetowhite lesion; acetowhite lesion occupying less than 75% of the transformation zone; acetowhite lesion amenable to complete coverage by the type of the cryoprobe; and lesions not suspicious of cancer [12]. Women who were diagnosed as VIA uncertain or suspicious of cancer were referred to the gynecologists for appropriate diagnosis and treatment. VIA positive women were scheduled for a post treatment follow up after six months, and VIA negative women were advised to rescreen within the next three years.

### VIA training and quality control

We trained seventeen nurses and six gynaecologists in the conduct of VIA screening. The training module included practical and theoretical sessions on the female reproductive anatomy; cervical cancer aetiology and prevention; informed consent process; communication and counselling skills; performing VIA and digital cervicography; use of data collection tools; data entry; and treatment with thermocoagulation. The training modules were based on cervical cancer recommendations from the WHO and VIA practical manuals from IARC [6,12]. During the practical training sessions nurses and doctors performed VIA under direct supervision of trainers. All trainees completed a pre and post assessment work up and were required to score at least 80% in the post assessment test before working in the screening clinic. Following the training courses, trainers worked with nurse providers in the screening clinic for at least one week. Training sessions were conducted prior to site activation, with refresher courses held after two years.

We implemented monthly Quality Assurance (QA) meetings to monitor provider performance and improve skills of providers except for a ten-month period between February 2012 and November 2012 when meetings could not be held for logistic reasons. These meetings were held at the central coordinating office at Abuja and were attended by all nurse providers. Nurse providers from sites close to the coordinating office attended in person while nurse providers from remote sites attended via video conferencing.

At each QA meeting, all images of the cervix (cervigraphs) taken before and after application of acetic acid during VIA screening were reviewed by a consultant gynecologist/colposcopist (YO). One nurse from each site would present the cervigraphs from that site, noting the nurse providers’ diagnosis and its rationale. Attendees would ask questions and finally the consultant gynecologist/colposcopist would announce his diagnosis for the cervigraph. Discordant diagnoses were highlighted and discussed, and clients with such diagnoses were recalled for appropriate management.

In addition to the monthly QA meetings, we also utilized a web-based application for real time consultation. In cases, where nurses wanted a second opinion, the nurses uploaded cervigraphs unto the web-based platform which immediately alerted collaborating gynecologists via SMS to provide a real-time diagnosis for the uploaded cervigraphs. Collaborating gynecologists working in each of the hospitals also provided on-site supervision.

### Data collection

Nurse providers collected data on demographics; documented findings from physical and abdominopelvic examinations; recorded findings from VIA assessments, details of treatment if provided and follow up plans. The nurse providers performed all VIA procedures and the gynecologist/colposcopists only made an assessment at the review meeting after cervigraphs had been discussed.

### Statistical analysis

For these analyses, we de-identified the screening clinics and referred to them as Sites A, B, C, D, and E. We were able to retrieve the quality assurance meeting reports for 31 months for Site A; 30 months for Site B; 7 months for Site C; 6 months for Sites D and E. All the nurses working at a given screening site were grouped together and analyses were conducted per site.

We calculated weighted kappa statistics to estimate the interobserver agreement for interpretations of cervigraphs between nurses and the consultant gynecologist/colposcopist by month and by site. We defined weights based on the following rationale: diagnosis of VIA positive and suspicious of cancer were reasonably similar; that VIA negative and uncertain are reasonably similar; but the VIA positive/suspicious of cancer group was not in any way similar to the VIA negative/uncertain group. We used Landis and Koch’s guidelines for the interpretation of kappa estimates where values below 0.0 are considered poor; 0.00–0.20 slight; 0.21–0.40 fair; 0.41–0.60 moderate; 0.61–0.80 substantial; 0.81–1.00 almost perfect [13]. In some months, all clients were negative hence the weighted kappa’s statistic could not be calculated for those months. We used univariate linear regression models to investigate site characteristics as predictors of agreement, using the mean weighted kappa statistic per site as the outcome variable. We did not perform further adjustments in multivariable regression models because of the limited number of sites.

We explored the temporal trends in agreement between nurse providers and the consultant gynecologist/colposcopist using two components—the overall trend in weighted kappa statistics over the study period and the monthly cyclical variation in weighted kappa statistics. We estimated the overall trend during the study period using linear regression models with time as the explanatory variable and weighted kappa statistic as the outcome variable for each site. We performed sensitivity analysis to investigate the trend in weighted kappa statistics for Sites A and B before and after the QA interruption by using linear piecewise regression models with splines at months 16 and 27, the period during which QA meetings were suspended.

We modelled monthly cyclical variation using autoregressive integrated moving (ARIMA) time series models. First, we used a time series filter, the compound, nonlinear, resistant, Hanning smoother to visually inspect the monthly cyclical patterns of the weighted kappa estimates over time. ARIMA model parameters were estimated using the Box Jenkins methodology, defined as *p*, *d*, and *q* [14]. In model specification, first we evaluated the trend component (*d*) for each site by performing Dicky Fuller tests [15]. As the data for both sites, A and B exhibited stationarity, we did not need to perform any further differentiation and *d* = 0 was used in our ARIMA models. Next, we evaluated autocorrelation functions (ACF) and partial autocorrelation functions (PACF) to identify the autoregressive (AR) component (value of *p*) and moving average (MA) component (value of *q*). We fitted several ARIMA models and performed model diagnostics, including residual plots and autocorrelation functions. We compared models using the Akaike Information Criteria (AIC) and selected the model with the lowest AIC value. Time series analysis were not performed for sites C, D, and E because of limited time series data. All statistical analyses were performed in Stata (Stata version 15).

### Ethical considerations

This study was reviewed and approved by the National Health Research Committee of Nigeria (NHREC protocol number: NHREC/01/01/2007-19-09-2014, NHREC approval number: NHREC/01/01/2007-25/09/2014). All participants provided a written informed consent.

## Results

### Participant and site characteristics

By May 2014, our VIA cervical cancer screening program was operational in five clinics around the country. Of these clinics, two were activated in 2010; two in 2012 and one in 2013 (Table 1). Sites A, B had five and four nurse providers respectively, while Sites C, D and E had two nurse providers each. At each site, there was at least one nurse provider who has a Bachelor’s degree in Nursing. Cumulative experience of the nurses in providing VIA screening ranged from 13 years in Site A to two years in Site C. All sites had a collaborating gynecologist on site, however, the level of engagement varied from an average of six clinic visits per month in site A to one clinic visit per month in Site E and Site D. The web based online consultation forum was most commonly used by nurse providers in Site A.

**Table 1:** Characteristics of VIA screening clinics.

Site characteristics	Site A	Site B	Site C	Site D	Site E
Year of activation	2010	2010	2013	2012	2012
Number of months fully operational by May 2014	44	44	13	9	9
Total number of nurses	5	4	2	2	2
Cumulative experience of nurses in performing VIA (years)	13	10	2	5	4
Average number of formal training sessions per nurse	2	2	1	2	1
Highest qualifications of nurses					
Diploma certificate, n (%)	1 (20)	1 (25)	1 (50)	1 (50)	0 (0)
Bachelor’s degree, n (%)	2 (40)	2 (50)	1 (50)	1 (50)	2 (100)
Master’s degree, n(%)	2 (40)	1 (50)	0 (0)	0 (0)	0 (0)
Engagement of onsite gynecologist (average number of visits to screening clinic per month)	6	3	2	1	1
Use of online consultation forum (average number of posts per month in the year 2013)	1	0	0	0	0
Site location	Central	Central	Remote	Central	Remote

Baseline characteristics of clients seen in the VIA screening program are provided in Table 2. Of a total of 4,961 clients seen by May 2014, majority were HIV positive (55%); married (62%); Christians (84%) and educated to post-secondary levels (55%). Baseline prevalence of precancerous lesions of the cervix as identified by a diagnosis of VIA positive was 6% in this population.

**Table 2:** Baseline characteristics of participants in the VIA screening program (2010–2014).

	A = 1979 n (%)	B = 1775 n (%)	C = 500 n (%)	D = 352 n (%)	E = 355 n (%)	Total = 4961 n (%)
Mean age ± SD, years	36 ± 7	36 ± 7	43 ± 9	37 ± 9	37 ± 9	37 ± 8
Mean sexual debut age ± SD, years	20 ± 4	19 ± 4	22 ± 4	21 ± 4	19 ± 4	20 ± 4
HIV status						
Positive	1278 (64)	1301 (73)	16 (3)	39 (11)	110 (31)	2744 (55)
Negative	271 (14)	310 (18)	453 (91)	308 (88)	238 (67)	1580 (32)
Unknown	430 (22)	164 (9)	31 (6)	5 (1)	7 (2)	637 (13)
Marital status						
Single	410 (21)	253 (14)	32 (6)	90 (26)	43 (12)	828 (17)
Married	1108 (56)	1042 (59)	427 (86)	222 (63)	269 (76)	3068 (62)
Widowed/Divorced/Separated	458 (23)	480 (27)	40 (8)	40 (11)	43 (12)	1061 (21)
Religion						
Christian	1722 (87)	1456 (82)	466 (94)	301 (86)	231 (65)	4176 (84)
Muslim	249 (13)	315 (18)	29 (6)	51 (14)	124 (35)	768 (16)
Highest formal education						
None	79 (4)	144 (8)	4 (1)	3 (1)	73 (21)	303 (6)
Primary	197 (10)	339 (19)	48 (10)	19 (5)	48 (14)	651 (13)
Secondary	500 (26)	541 (31)	105 (21)	46 (13)	69 (19)	1261 (26)
Post-secondary	1186 (60)	732 (42)	341 (68)	281 (81)	164 (46)	2704 (55)
VIA diagnosis by nurses						
VIA negative	1818 (92)	1668 (94)	453 (91)	326 (93)	347 (98)	4632 (93)
VIA uncertain	6 (0)	4 (0)	8 (2)	0 (0)	1 (0)	19 (1)
VIA positive	149 (8)	99 (6)	38 (8)	19 (5)	6 (2)	311 (6)
VIA suspicious of cancer	3 (0)	1 (0)	1 (0)	6 (2)	1 (0)	12(0)

Abbreviations: VIA–Visual inspection with acetic acid; SD–Standard deviation.

**Fig 1:**
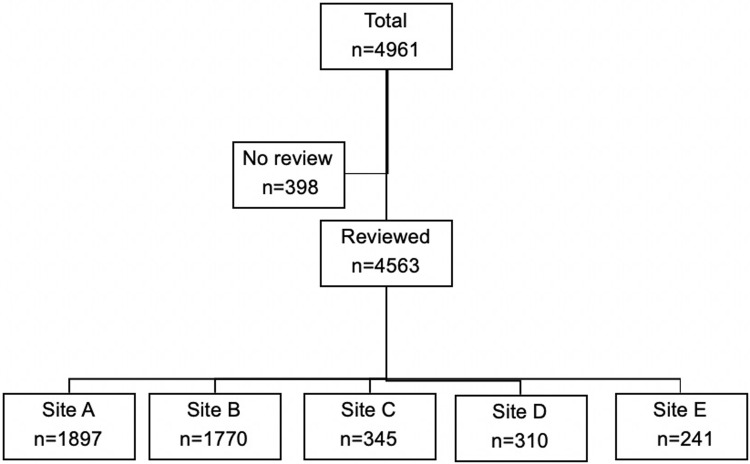
Participant flow chart.

Of the 4,961 clients seen in the VIA screening program, cervigraphs of 4,602 clients were reviewed by the gynecologist/colposcopist at the monthly QA meetings. Details of the distribution of the reviewed cervigraphs by site are provided in Fig 1.

### Site characteristics as predictors of agreement

Weighted kappa estimates for the duration under study were almost perfect for site D (0.89); substantial for sites A and B (0.78 and 0.73 respectively); moderate for site E (0.50); and fair for Site C (0.34) (Table 3).

**Table 3:** Mean weighted kappa estimates by year and site: 2010 to 2014.

	Year 1 (Oct–Dec 2010) Weighted kappa		Year 2 (Jan–Dec 2011) Weighted kappa		Year 3 (Jan–Dec 2012) Weighted kappa		Year 4 (Jan–Dec 2013) Weighted kappa		Year 5 (Jan–May 2014) Weighted kappa		Total Year 1–5 Weighted kappa	
Site*	n	Mean (Range)	n	Mean (Range)	n	Mean (Range)	n	Mean (Range)	n	Mean (Range)	n	Mean (Range)
A	3	0.92 (0.86–100)	10	0.93 (0.84–100)	2	0.70 (0.67–0.72)	12	0.71 (0.00–100)	4	0.00 (0.00–0.00)	31	0.78 (0–100)
B	3	0.59 (0.12–0.97)	10	0.76 (0.16–100)	1	0.87 (0.87–0.87)	12	0.72 (0.05–100)	4	0.79 (67–95)	30	0.73 (0.05–100)
C	-	-	-	-	-	-	5	0.36 (0.00–100)	2	0.27 (0.0–0.54)	7	0.34 (0.00–100)
D	-	-	-	-	-	-	4	0.95 (0.79–100)	2	0.64 (0.64–0.64)	6	0.89 (0.64–100)
E	-	-	-	-	-	-	2	0.50 (0.00–100)	4	0.50 (0.00–100)	6	0.50 (0.00–100)

*Sites C, D and E were not operational in 2010–2012.

Mean weighted kappa estimate is provided per site for each year the site was fully functional in providing cervical cancer screening services. Sites C, D and E were not operation in 2010, 2011 or 2012, therefore they do not have data for those years. n refers to the number of quality assurance reports available per site per year. The mean weighted kappa is the average of the weighted kappa per site for a given year with the range of weighted kappa estimates provided in parenthesis. Total provides a summary estimate per site for all the years the site was operational.

In univariate linear regression models, site characteristics that were significantly associated with improved weighted kappa statistics were attendance at more formal VIA training sessions (β = 0.47, 95% CI: 0.02–0.93, p = 0.04); high level of engagement by onsite gynecologist (β = 0.11, 95% CI: 0.01–0.21, p = 0.04) (Table 4). Remote sites had significantly less agreement than sites which were in close proximity to the coordinating site (β = -0.47, 95% CI: -0.92–0.02, p = 0.04). There was no association between agreement and the number of months during which a site had been operational (β = 0.01, 95% CI: -0.01–0.03, p = 0.29), or the cumulative experience of nurse providers (β = 0.04, 95% CI: -0.03–0.12, p = 0.19).

**Table 4:** Site characteristics as predictors of weighted kappa estimates from univariate linear regression models.

Variable	Average change per unit increase (β)	95% CI	p value
Number of months operational	0.01	-0.01–0.03	0.29
Number of nurses (n)	0.11	-0.17–0.40	0.35
Experience (years)	0.04	-0.03–0.12	0.19
Number of formal VIA training sessions	0.47	0.02–0.93	0.04
Number of nurses with Bachelor’s degree or higher	0.07	-0.29–0.43	0.61
Onsite gynecologist engagement	0.11	0.01–0.21	0.04
Distance from site			
Central	1.00 (Reference)		
Remote	-0.47	-0.92–0.02	0.04

### Trends in agreement over time

For every additional month of site being fully operational, the weighted kappa statistic, comparing nurse providers at the site and the consultant gynecologist/colposcopist, increased by 0.21 (95% CI: -0.62–1.05, p = 0.60) for Site B and 1.03 (95% CI: -42.4–44.5, p = 0.93) for Site E; and decreased for three other sites (β = -0.96, 95% CI: -1.79–0.13, p = 0.03 for Site A; β = -2.90, 95% CI: -11.7–5.90, p = 0.44 for Site C; and β = -5.10, 95% CI: -17.8–7.54, p = 0.30 for Site E). These changes were only significant for Site A (Table 5).

**Table 5:** Average change in weighted kappa estimate per month for five sites: 2012–2014.

Site	Number of QA monthly reports available (n)	Predicted weighted kappa at site activation (%)	Average change in weighted kappa per month (β)	95% CI	p value
Site A	31	98	-0.96	-1.79–0.13	0.03
Site B	30	68	0.21	-0.62–1.05	0.60
Site C	7	53	-2.90	-11.7–5.90	0.44
Site D	6	99	-5.10	-17.8–7.54	0.30
Site E	6	45	1.03	-42.4–44.5	0.93

**Fig 2:**
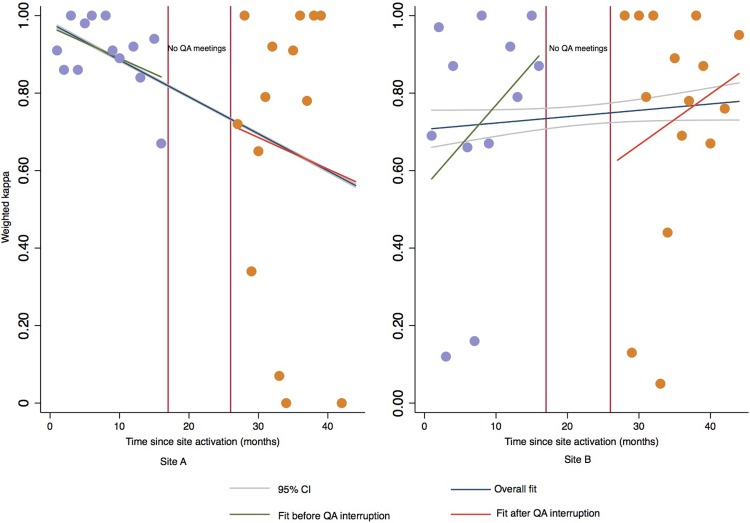
Sensitivity analysis comparing kappa estimates from linear piecewise regression models with splines and linear regression models without splines. The period of QA interruption is indicated by red vertical lines. Average change in weighted kappa estimates with each additional month a site is fully operational before (Fit before QA interruption) and after (Fit after QA interruption) QA interruption are provided as well as the overall change in weighted kappa estimate ignoring the QA interruption (Overall fit) with confidence intervals (95% CI). In Site A, there was a decrease in weighted kappa estimates both before and after the interruptions in QA meetings. In contrast, there was increasing trend in weighted kappa estimates for Site B, both before and after the QA interruption. Results from the more parsimonious linear regression models (overall fit) were not significantly different from the models with splines.

In sensitivity analysis, weighted kappa statistics declined with each additional increase in month of being operational in the periods before (β = -0.81, 95% CI: -4.35–2.74, p = 0.64) and after (β = -0.81, 95% CI: -4.63–3.00, p = 0.66) QA interruption for Site A (Fig 2). However, these declining trends were not significant. In contrast, weighted kappa statistics in Site B increased over time in the periods before (β = 2.12, 95% CI: -1.59–5.82 p = 0.23) and after (β = 1.32, 95% CI: -2.16–4.79, p = 0.64) QA interruptions, these trends were also not significant (Fig 2). Fig 2 provides a graphical comparison of coefficient estimates from the linear piecewise regression models from sensitivity analysis and the more parsimonious linear regression models per site.

### Monthly cyclical variation

Monthly kappa estimates for site A for the period under study were not serially correlated (ρ_1_ = -0.11, p = 0.54) indicating that the data in this series were random and independent. Therefore, further ARIMA models were not considered for Site A.

**Fig 3:**
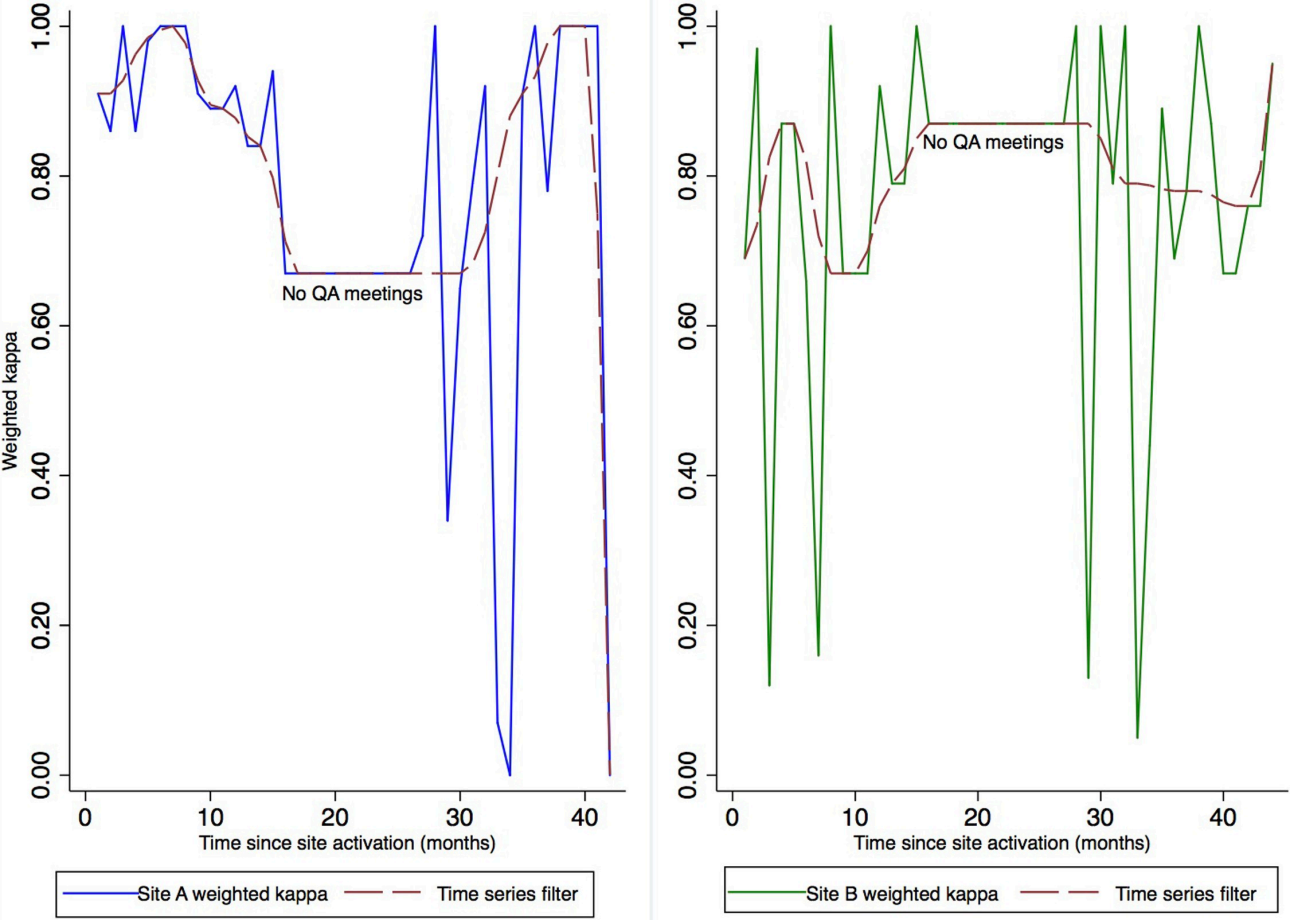
Variability in monthly weighted kappa statistic for Sites A and B: 2012–2014. Weighted kappa estimates for Site A were highly variable, with no correlations over time, indicating that results for each month were random and independent of results from previous months. In Site B, monthly weighted kappa estimates demonstrated an autoregressive behavior such that an estimate above the mean in month *(t-1)* was followed by an estimate below the mean in the next month *(t)*.

For Site B, our data displayed a significant autoregressive behavior for the first order ARIMA model (1,0,0). The weighted kappa statistics per month at this site were slightly autocorrelated (ρ_1_ = -0.40, p = 0.03). The lagged change in weighted kappa estimate by one month (*t -1*) was a significant predictor of expected kappa estimate for the current month *(t)* (AR1 term = -0.40, 95% CI: -0.71–0.09, p = 0.01). As the coefficient of the AR1 term was negative, monthly kappa estimates were highly variable and oscillatory such that an estimate above the mean in month *t-1* was followed by an estimate below the mean in month *t*. Fig 3 provides a graphical representation of the oscillatory nature of kappa estimates by site.

## Discussion

In this study, we found that regular onsite supervision, more formal VIA training sessions and proximity to the cervical cancer screening coordinating site were associated with a higher diagnostic agreement between nurse providers and consultant gynecologist/colposcopist. Overall, there were no significant increases in agreement over time and there was significant monthly oscillatory variation in agreement for one of two sites evaluated. While existing recommendations suggest that robust quality assurance and improvement measures such as regular supervision and training can improve VIA screening programs [5], our results suggest these may not be enough to improve the diagnostic accuracy of nurse providers over time.

Our finding of lack of association between the duration of screening operations at specific sites, cumulative experience of the nurse providers and diagnostic agreement is consistent with the results from a screening study of 36,000 women in the Amazonian Peru, in which midwives performed VIA screening [16]. In that study, experience was measured by the number of VIA tests performed and the investigators found that regardless of the number of VIA tests performed, VIA results were highly variable. These findings contrast with results from studies that suggest a learning period of a few weeks to a few months are sufficient to acquire proficiency [11, 17]. However, it must be noted that the indices used to measure proficiency in these studies are different, and a meta-analysis which uses identical indices may be more informative. For example, in Cameroon, DeGregorio *et al*. indicated that proficiency was measured by the ability of trainee nurse providers to pass a written exam and demonstrate competence in performing VIA based screening procedures [17]. All the nurse providers in our program scored over 80% in our post training assessment, if we used post training assessment as our metric for ascertaining proficiency, all of our nurse providers would have been considered proficient in the first month of site activation.

Despite the use of several quality improvement measures in our study, there was a high temporal variability in agreement between the nurse providers and the consultant gynecologist/colposcopists. We did not identify any previous study in the literature that provided temporal estimates of interobserver agreement between nurse providers and physicians for VIA screening. However, Almonte *et*. *al*. found significant temporal variability in the proportion of VIA screening examinations that were classified as positive which was independent of the level of experience of the nurse provider [16].

We observed that trends in the diagnostic capabilities of nurse providers over time varied by site. In some sites, the trend was convergent while other sites exhibited a divergent trend. It is possible that site characteristics which are proxies for intensive supervision such as distance from coordinating centre, onsite gynaecologist engagement and nature of gynaecologist involvement may account for some of the differences in trends observed. It is also notable that sites which had high weighted kappa estimates at site initiation were more likely to exhibit a decline in weighted kappa over time than sites which had low kappa estimates at the time of site activation. It is plausible that onsite gynaecologist supervision was reduced when the initiation kappa estimates were high and conversely increased when site initiation kappa estimates were low leading to the variability in trends that we observed in our data.

In our study, we were able to characterize VIA performance over a relatively long period with a moderately sized population and include clinics from different regions of Nigeria. Opportunistic screening using VIA protocols have been implemented by several groups in Nigeria, however this is the first systematic evaluation of its performance over time in Nigeria and Sub-Saharan Africa [18–20].

We were unable to assess the agreement between individual nurse providers and the consultant gynecologist/colposcopist as we only collected aggregate data per site per month. It is conceivable that agreement would vary by individual provider characteristics. It would be interesting to identify specific provider characteristics that are associated with better performance and use this to tailor recruitment and training needs for VIA based cervical cancer screening clinics. Nevertheless, since the nurses worked collaboratively at the sites, we believe that analyzing them as a group is appropriate. The providers also provided peer education and mentoring to each other which is beneficial for quality improvement purposes.

We did not blind our consultant gynecologist/colposcopist to the diagnosis of the nurse providers. It is possible that the diagnosis made by the nurses may have influenced the impressions made by the gynecologist/colposcopist. The effect of this potential bias would be an increase in the weighted kappa estimates. Therefore, our estimates would be conservative at best and the true performance of VIA screening may be worse.

The use of weighted kappa statistics to measure agreement has some inherent limitations. The kappa statistics can be low or inestimable even when there is high percent agreement if the prevalence of traits is low and when marginal totals are asymmetric [21]. If all diagnosis in a given month are VIA negative and both nurse providers and the consultant gynecologist/colposcopist are in perfect agreement, the weighted kappa statistic would still be inestimable due to the asymmetric margin totals. This paradox provides some explanation for some of the large dips in interobserver agreement observed in our study.

## Conclusion

Our results show that even with considerable investments in quality improvement, there was no improvement in diagnostic accuracy of nurse led VIA screening with cervicography over time and regular intense supervision was required to maintain a level of quality in our screening program. Policy makers in LMIC should take the result of this and similar studies into consideration in selecting appropriate cervical cancer screening strategies for their populations.

## Supporting information

S1 File Monthly kappa estimates by site. Dataset of kappa statistics on which we performed our analysis.
(XLS)
